# Investigation into pathophysiology of naturally occurring palatal instability and intermittent dorsal displacement of the soft palate (DDSP) in racehorses: Thyro-hyoid muscles fatigue during exercise

**DOI:** 10.1371/journal.pone.0224524

**Published:** 2019-10-25

**Authors:** Marta Cercone, Emil Olsen, Justin D. Perkins, Jonathan Cheetham, Lisa M. Mitchell, Norm G. Ducharme

**Affiliations:** 1 Department of Clinical Sciences, College of Veterinary Medicine, Cornell University, Ithaca, New York, United States of America; 2 Department of Clinical Sciences and Services, Royal Veterinary College, London, Hatfield, United Kingdom; Massey University, NEW ZEALAND

## Abstract

Exercise induced intermittent dorsal displacement of the soft palate (DDSP) is a common cause of airway obstruction and poor performance in racehorses. The definite etiology is still unclear, but through an experimental model, a role in the development of this condition was identified in the dysfunction of the thyro-hyoid muscles. The present study aimed to elucidate the nature of this dysfunction by investigating the spontaneous response to exercise of the thyro-hyoid muscles in racehorses with naturally occurring DDSP. Intramuscular electrodes were implanted in the thyro-hyoid muscles of nine racehorses, and connected to a telemetric unit for electromyographic monitoring implanted subcutaneously. The horses were recruited based on upper airway function evaluated through wireless endoscopy during exercise. Five horses, with normal function, were used as control; four horses were diagnosed as DDSP-affected horses based on repeated episodes of intermittent dorsal displacement of the soft palate. The electromyographic activity of the thyro-hyoid muscles recorded during incremental exercise tests on a high-speed treadmill was analyzed to measure the mean electrical activity and the median frequency of the power spectrum, thereafter subjected to wavelet decomposition. The affected horses had palatal instability with displacement on repeated exams prior to surgical implantation. Although palatal instability persisted after surgery, only two of these horses displaced the palate after instrumentation. The electromyographic traces from this group of four horses showed, at highest exercise intensity, a decrease in mean electrical activity and median power frequency, with progressive decrease in the contribution of the high frequency wavelets, consistent with development of thyro-hyoid muscle fatigue. The results of this study identified fatigue as the main factor leading to exercise induced palatal instability and DDSP in a group of racehorses. Further studies are required to evaluate the fiber type composition and metabolic characteristics of the thyro-hyoid muscles that could predispose to fatigue.

## Introduction

Horses are remarkable athletes whose performances are highly affected by their respiratory system function [1]. The horse is an obligate nasal breather, where the caudal free border of the soft palate seals the nasopharynx ventrally to the epiglottis [2]; any perturbation of this normal anatomical relationship causes increased airflow turbulence and respiratory impedance [3]. A specific condition whereby the free caudal border of the soft palate moves dorsal to the epiglottis during exercise, named intermittent Dorsal Displacement of the Soft Palate (DDSP), has been recognized as a common cause of airway obstruction in racehorses with a reported prevalence of 10–20% in horses with poor performance [4–7].

The definitive cause of naturally occurring DDSP is still uncertain. Three potential etiologies have been proposed based on experimental work: dysfunction of the pharyngeal branch of the vagus nerve affecting the levator veli palatini, palatinus and palatopharyngeus muscles [8], dysfunction of the hypoglossal nerves affecting the styloglossus, genioglossus, geniohyoid and hyoglossus muscles [9], and dysfunction of the thyro-hyoid (TH) muscles [10]. The current standard surgical treatment for DDSP (laryngeal tie-forward) was developed to move the larynx to a more rostral and dorsal position and correct a dysfunction of the TH muscle [11].

The TH are paired flat muscles located on each side of the larynx, extending from the ipsilateral caudal border of the thyro-hyoid bone to the lateral surface of the lamina of the thyroid cartilage [2]. From their anatomical location, it has been proposed that the function of the TH muscles is to draw the larynx forward and the basihyoid caudally [12]. Physiological studies in dogs at rest have shown that the TH muscles move the larynx rostrally [13]. The effect of TH muscle contraction in horses was evaluated using electrical stimulation after hypoglossal nerve block: Bilateral thyro-hyoid muscle stimulation induced significant laryngeal elevation from a cervical to a more dorso-rostral position [14]. Therefore, it appears that dysfunction of the TH muscles results in a more caudal and ventral position of the larynx relative to the basihyoid bone, and a more ventral position relative to the soft palate.

It is however unknown if and why TH muscle dysfunction develops in naturally-occurring disease. As DDSP is an exercise-associated condition, elucidating the physiologic response of the TH muscles to exercise and investigating if this response is altered in horses affected by this condition, can provide knowledge on the pathogenesis of DDSP, so helping in designing preventive measures and alternative treatments. Electromyography (EMG) allows to investigate muscle recruitment, degree of activation and fatigue, through measurement of the EMG signal amplitude and power spectrum [15]. The EMG power density spectrum, not only reflects motor unit recruitment and conduction velocity, but provides information on fiber type proportion of the muscle, showing a higher median power frequency (> 150Hz) in muscles with a greater percentage of glycolytic fibers (type II) [16,17]. A specific electromyographic pattern has been associated with muscle fatigue, characterized by a decrease in the signal amplitude and a decrease in median power frequency [18,19].

We know that TH muscle electromyographic (EMG) activity, as well as other upper airways muscles, increases with increasing exercise intensity in horses with normal airway function [20–22], but the EMG activity of the TH muscles in horses affected by DDSP has not previously been investigated. The purpose of this study was to evaluate and compare the spontaneous electromyographic activity of the TH muscle during increasing exercise intensity in control horses, not affected by DDSP, and horses naturally affected by DDSP. Specifically, we wanted to 1) assess TH muscle fatigue based on EMG; utilizing multiple techniques including EMG amplitude, spectral analysis and time-frequency domain analysis (wavelet decomposition) [23]; and 2) perform a cluster analysis to assess the presence of low versus high frequency band clusters and their contribution to muscle activation during exercise for both control and DDSP-affected horses.

The present study determined the TH muscle response to incremental exercise in the horse and, identifying the relative contribution of low- and high-frequency band clusters to the EMG spectrum, enlightened novel aspects of the palatal instability and DDSP etiology, laying the basis for a new approach in the management of this condition in racehorses.

## Materials and methods

This study was performed in accordance with the PHS Policy on Humane Care and Use of Laboratory Animals, federal and state regulations, and was approved by the Institutional Animal Care and Use Committees (IACUC) of Cornell University (Protocol Number: 2016–0027) and the Ethics and Welfare Committee at the Royal Veterinary College (Project License number: PPL70/7122).

### Study design

Adult horses belonging to the research herds of the two institutions were recruited to the study if in good health and following evaluation of the upper airway through endoscopic exam, at rest and during exercise, either overground or on a high-speed treadmill using a wireless videoendoscope. The institution ethics committees granted the consents to perform procedures on these animals. Horses were categorized as “DDSP” affected horses if they presented with palatal instability AND exercise-induced intermittent dorsal displacement of the soft palate consistently during multiple (n = 3) exercise tests, or “control” horses if they did not experience dorsal displacement of the soft palate during exercise and had no DDSP nor palatal instability during exercise, soft palate or sub-epiglottic ulcerations. After enrollment in the study, the horses were brought into one of two research facilities and given a 7-day acclimatization period prior to any procedure. Daily record logs of medical procedures were maintained. Horses were trained on a high-speed treadmill for sequential evaluations of their upper airway function during incremental exercise. Horses were then instrumented with bipolar or quadripolar electrodes, in one or both TH muscles for EMG recording, hard wired to a wireless transmitter for remote recording implanted in the proximal cervical area. EMG recordings were then made during an incremental exercise test based on the percentage of maximum heart rate (HR_max_). At the end of the study, the horses were enrolled in other research programs.

#### Surgical instrumentation

**Fig 1:**
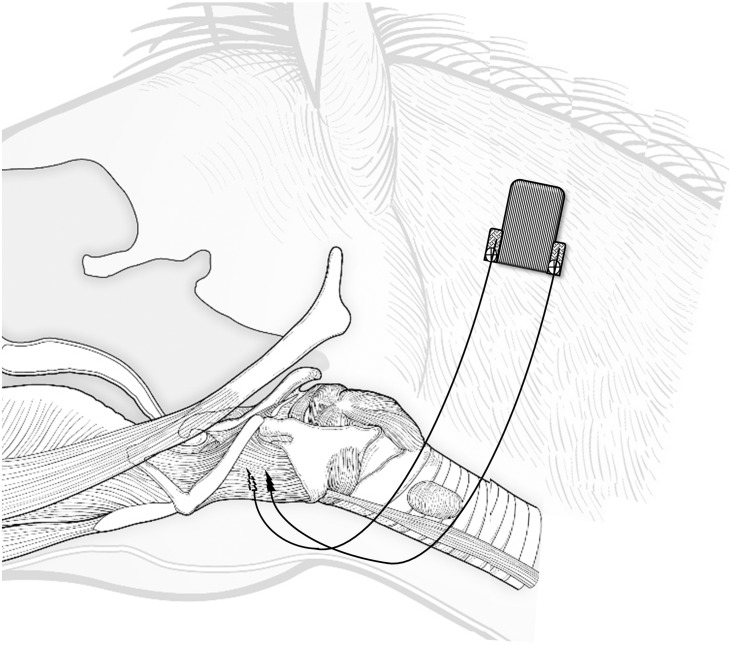
Schematic illustration to show the position of the intramuscular electrodes and electromyography transmitter. The diagram illustrates the left side of the horse, and the electrodes within the two thyro-hyoid muscles. The electrodes leads are tunneled subcutaneously and connected to the electromyography transmitter implanted subcutaneously over the left proximal neck.

Horses were maintained under general inhalation anesthesia in dorsal recumbency. After aseptic preparation of the ventral laryngeal area, a longitudinal 15-cm midline incision was made from 5 cm rostral to the basihyoid bone to 5 cm caudal to the cricoid cartilage. Blunt dissection was performed to expose the ventral and ventro-lateral aspect of the TH muscles and electrodes with a spiraling cathode at their tip (KY-5 or K5-P4, OSYPKA, Rheinfelden, Germany; or customized 3fr bipolar electrodes, MED-EL, Innsbruck, Austria) were positioned in one or both TH muscles using an insertion cannula (18G needle, Pajunk GmbH, Geisingen, Germany). The electrode wires were secured to their respective muscles with sutures over a protective electrode cuff, and then tunneled subcutaneously to reach a telemetry transmitter implanted in the left proximal cervical area (Fig 1). Two types of transmitters were used (details in the EMG recording paragraph). All horses received broad-spectrum antimicrobials (trimethoprim-sulfamethoxazole; 30 mg/kg bwt, PO, q 12 h) and the non-steroidal anti-inflammatory drug phenylbutazone (1 mg/kg bwt, PO, q 12 h) for 5–7 days after surgery. Horses were examined daily for evidence of complications or illness (signs of pain, swelling, or dysphagia) and allowed a period of 2 weeks after surgery before resumption of treadmill exercise.

#### Training protocol

Prior to instrumentation, the horses were gradually acclimated to exercise on treadmill, starting with walk and trot speed and, within 1 to 2 weeks from the first acclimation session, depending on their physical fitness, introduced to canter and gallop speed. When the horses were considered adequately fit, they were started on a training protocol that included exercise performed 5 days/week for a minimum of 28 km/week. Endurance training was performed at 4m/sec (trot) for about 3–5 km daily. Three times/week, bouts of high-speed intervals were added to the endurance training: 1200 m at 8m/sec (canter) twice/week, and 800 m at 10m/sec speed (slow gallop) once/week. Following surgical instrumentation, the horses were kept at rest for 2 weeks, and then gradually resumed the training on treadmill. After a minimum of 4 continuative weeks at full training, they underwent the incremental tests with emg and endoscopy recording.

#### Incremental Standardized Exercise Test (SET)

After surgical instrumentation and 1-month full training on treadmill, each horse performed the first 4-step incremental SET while recording TH electromyographic activity, heart rate, upper airway videoendoscopy, pharyngeal airway pressures, and gait frequency measurements. Horses were evaluated at exercise intensities corresponding to 50, 80, 90 and 100% of their maximum heart rate with each speed maintained for 1 minute [24]. Determination of maximal heart rate (HR_max_) was performed using a standardized exercise protocol. During the HR_max_ trial the treadmill was started at time 0, accelerated to 4 m/sec and held at this speed for 4 minutes; then accelerated to 6 m/sec and kept at that speed for 1 min. Each subsequent minute, the treadmill was accelerated by 1 m/sec until the heart rate showed no further increase and/or the horse was no longer capable of maintaining position near the front of the treadmill [10]. The heart rate at this speed was determined to be the HR_max_, and the speed corresponding to the 50, 80, 90% of HR_max_ were derived based on the regression line between the speed and corresponding heart rate recorded during the trial. Horses were provided a rest period of at least 3 days to allow recovery before any subsequent exercise test.

Laryngeal function during the incremental SET was recorded using a wireless videoendoscope (Optomed, Les Ulis, France), which was placed into the nasopharynx via the right ventral nasal meatus. Nasopharyngeal pressure was measured using a Teflon catheter (1.3 mm ID, Neoflon) inserted through the left ventral nasal meatus to the level of the left guttural pouch ostium. The catheter was attached to differential pressure transducers (Celesco LCVR, Celesco Transducers Products, Canoga Park, CA, USA) referenced to atmospheric pressure and calibrated from -70 to 70 mmHg [25,26]. Occurrence of episodes of dorsal displacement of the soft palate was recorded and number of swallows during each SET were counted for each speed interval because in prior work, swallowing has been shown to increase immediately prior to DDSP [27].

#### EMG recording

EMG data was recorded through a wireless transmitter device implanted subcutaneously (Fig 1). Two different transmitters were used: 1) TR70BB (Telemetry Research Ltd, Auckland, New Zealand) with 12bit A/D conversion resolution, AC coupled amplifier, -3dB point at 1.5Hz, 2KHz sampling frequency (n = 5 horses); or 2) ELI (Center for Medical Physics and Biomedical Engineering, Medical University of Vienna, Vienna, Austria) [28], with 12bit A/D conversion resolution, AC coupled amplifier, amplifier gain 1450, 1KHz sampling frequency (n = 4 horses). The EMG signal was transmitted through a receiver (TR70BB) or Bluetooth (ELI) to a data acquisition system (PowerLab 16/30—ML880/P, ADInstruments, Bella Vista, Australia). The EMG signal was amplified with octal bio-amplifier (Octal Bioamp, ML138, ADInstruments, Bella Vista, Australia) with a bandwidth frequency ranging from 20–1000 Hz (input impedance = 200 MV, common mode rejection ratio = 85 dB, gain = 1000), and transmitted to a personal computer. All EMG and pharyngeal pressure signals were collected at 2000 Hz rate with LabChart 6 software (ADInstruments, Bella Vista, Australia) that allows for real-time monitoring and storage for post-processing and analysis.

#### EMG signal processing

Electromyographic signals from the TH muscles were processed using two methods: 1) a classical approach to myoelectrical activity and median frequency and 2) wavelet decomposition. For both methods, the beginning and end of recording segments including twenty consecutive breaths, at the end of each speed interval, were marked with comments in the acquisition software (LabChart). The relationship of EMG activity with phase of the respiratory cycle was determined by comparing pharyngeal pressure waveforms with the raw EMG and time-averaged EMG traces.

For the classical approach, in a graphical user interface-based software (LabChart), a sixth-order Butterworth filter was applied (common mode rejection ratio, 90 dB; band pass, 20 to 1,000 Hz), the EMG signal was then amplified, full-wave rectified, and smoothed using a triangular Bartlett window (time constant: 150ms). The digitized area under the time-averaged full-wave rectified EMG signal was calculated to define the raw mean electrical activity (MEA) in mV.s. Median Power Frequency (MF) of the EMG power spectrum was calculated after a Fast Fourier Transformation (1024 points, Hann cosine window processing).

For the wavelet decomposition, the whole dataset including comments and comment locations was exported as .mat files for processing in MATLAB R2018a with the Signal Processing Toolbox (The MathWorks Inc, Natick, MA, USA). A custom written automated script based on Hodson-Tole & Wakeling [29] was used to first cut the .mat file into the selected 20 breath segments and subsequently process each segment. A bank of 16 wavelets with time and frequency resolution optimized for EMG was used. The center frequencies of the bank ranged from 6.9 Hz to 804.2 Hz [30]. The intensity was summed (mV^2^) to a total, and the intensity contribution of each wavelet was calculated across all 20 breaths for each horse, with separate results for each trial date and exercise level (80, 90, 100% of HR_max_ as well as the period preceding episodes of DDSP). To determine the relevant bandwidths for the analysis, a Fast Fourier transform frequency analysis was performed on the horses unaffected by DDSP from 0 to 1000 Hz in increments of 50Hz and the contribution of each interval was calculated in percent of total spectrum as median and interquartile range. According to the Shannon-Nyquist sampling theorem, the relevant signal is below ½ the sample rate and because we had instrumentation sampling either 1000Hz and 2000Hz we choose to perform the frequency analysis up to 1000Hz. The 0–50Hz interval, mostly stride frequency and background noise, was excluded from further analysis. Of the remaining frequency spectrum, we included all intervals from 50–100Hz to 450–500Hz and excluded the remainder because they contributed with less than 5% to the total amplitude.

#### Data analysis

At the end of each exercise speed interval, twenty consecutive breaths were selected and analyzed as described above. To standardize MEA, MF and mV^2^ within and between horses and trials, and to control for different electrodes size (i.e. different impedance and area of sampling), data were afterward normalized to 80% of HR_max_ value (HR_max80_), referred to as normalized MEA (nMEA), normalized MF (nMF) and normalized mV^2^ (nmV^2^). During the initial processing, it became clear that the TH muscle is inconsistently activated at 50% of HRmax and that speed level was therefore excluded from further analysis. The endoscopy video was reviewed and episodes of palatal displacement were marked with comments. For both the classical approach and wavelet analysis, an EMG segment preceding and concurrent to the DDSP episode was analyzed. If multiple episodes were recorded during the same trial, only the period preceding the first palatal displacement was analyzed. In horses that had both TH muscles implanted, the average between the two sides was used for the analysis. Averaged data from multiple trials were considered for each horse.

Descriptive data are expressed as means with standard deviation (SD). Normal distribution of data was assessed using the Kolmogorov-Smirnov test and quantile-quantile (Q-Q) plot. To determine the frequency clusters in the EMG signal, a hierarchical agglomerative dendrogram was applied using the packages Matplotlib, pandas, numpy and scipy in python (version 3.6.6) executed through Spyder (version 3.2.2) and Anaconda Navigator. Based on the frequency analysis, wavelets included in the cluster analysis were 92.4 Hz, 128.5 Hz, 170.4 Hz, 218.1 Hz, 271.5 Hz, 330.6 Hz, 395.4 Hz and 465.9 Hz. The number of frequency clusters was set to two based on maximum acceleration in a scree plot and maximum vertical distance in the dendrogram.

For continuous outcome measures (number of swallows, MEA, MF, and mV^2^) a mixed effect model was fitted to the data to determine the relationship between the outcome variable and relevant fixed effects (breed, sex, age, weight, speed, group) using horse as a random effect. Paired t-test was conducted to investigate side to side difference in the horses with bilateral implants. Tukey’s post hoc tests and linear contrasts used as appropriate. Statistical analysis was performed using JMP Pro13 (SAS Institute, Cary, NC, USA). Significance set at P < 0.05 throughout.

## Results

### Horses

A total of 9 racehorses were recruited into the study (mean age 4.3 ± 1.3 years, mean weight 504.4 ± 40.3 Kg). Four Thoroughbred horses (1 female, 3 castrated males) were categorized as DDSP-affected horses, while five horses (3 Standardbred, 2 Thoroughbred; 3 females, 2 castrated males) were considered control. No significant difference in age and weight was found between groups. All horses were instrumented with bipolar (or quadripolar) intra-muscular electrodes in one or both TH muscles (5 Fr electrodes in 5 horses; 3 Fr electrodes in 4 horses), connected to an electromyography transmitter implanted over the left proximal neck.

### Incremental standardized exercise tests

After instrumentation, a complete set of data could be obtained from all horses for at least one incremental SET. These tests were repeated up to 5 times. Technical difficulty (EMG trace quality, electrodes and/or EMG transmitter failure), made not possible to repeat the test successfully in all horses. Data used for the analysis derived from one test in 5 horses (4 control horses, 1 DDSP horse), from 3 to 5 tests in four horses (one control and 3 DDSP-affected horses). No significant difference was detected regarding the highest speed reached by each group during the tests, with a mean of 11.26 ± 1.04 m/s (p>0.05). None of the horses considered normal developed displacement of the soft palate nor palatal instability during any of the high-speed treadmill tests. Of the four horses diagnosed as DDSP-affected in exercise endoscopy prior to electrodes implantation, two demonstrated palatal instability followed by palatal displacement within the HR_max90_ and HR_max100_ intervals. DDSP episodes lasted >10seconds and resolved completely during release from the maximum exercise intensity phase. The other two horses had palatal instability but no episodes of palatal displacement after instrumentation. As previous studies suggested that palatal instability (PI) and DDSP are manifestations of the same condition, and PI represents the preliminary stage of a disorder that may progress to DDSP, as part of a syndrome named palatal dysfunction [5,31,32], we decided to maintain the four horses within the original group identified at the time of enrollment (DDSP).

#### Swallows

All horses had a non-significant higher number of swallows at low speed compared to higher speed. Mixed effect model identified a statistically significant effect of speed (p<0.0001) on the number of swallows made during exercise. The DDSP group showed a significant although weak negative linear correlation between swallows and speed: Swallows (n) = − 0.31*Speed (m/s) + 5.32, r = 0.50, 95% CI [0.26, 0.678], p = 0.0002.

#### Mean electrical activity (MEA)

**Fig 2:**
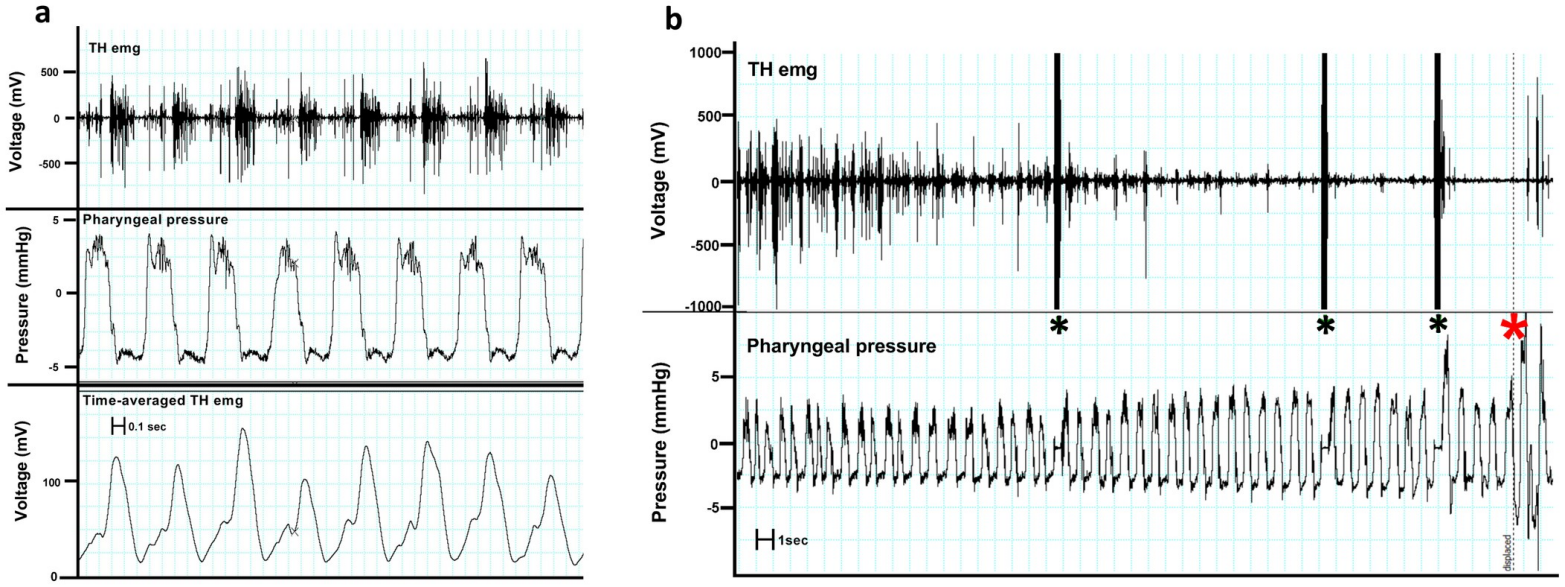
Electromyographic activity of the thyro-hyoid (TH) muscle in an exercising horse. TH electrical activity (TH emg) is synchronous with inspiration (2a), increases with swallowing and is absent immediately prior to dorsal displacement of the soft palate (DDSP) (2b). 2b) Representative raw electromyographic activity of the thyro-hyoid muscle and pharyngeal pressure in a horse affected with intermittent DDSP. Black asterisks indicate swallows; the red asterisk indicates the DDSP episode. Note the marked reduction in the breathing related EMG activity prior to development of DDSP. The Y-axis is limited to +/-1000mV to facilitate viewing of EMG trace.

Electromyographic recording of the spontaneous TH muscle activity showed minimal to no evident activity at rest and at low speed exercise (except during swallowing). During more intense exercise, the TH muscle showed inspiratory-related phasic activity (Fig 2a), starting at end-expiration and progressively increasing to peak at inspiration. The TH muscle EMG activity showed a gradual decrease before the episodes of palatal displacement (Fig 2b).

The mixed effects model identified a significant effect on MEA of group (p = 0.0106), with a significant interaction between groups (DDSP vs Control) and exercise intensity (p = 0.0314). No significant effect of age, sex, breed and weight was found (p>0.07). MEA showed a significant moderate positive linear correlation with speed in the control horses: MEA (mv.s) = -0.52 + 0.47*speed (m/s), r = 0.57, 95% CI [0.248, 0.776], p = 0.0016. In contrast, the DDSP horses showed a significant moderate quadratic correlation between speed and MEA: MEA (mv.s) = 10.55–0.02*speed (m/s) − 0.25*(speed (m/s) − 8.36)^2, r = 0.65, 95% CI [0.452, 0.78], p<0.0001.

**Fig 3:**
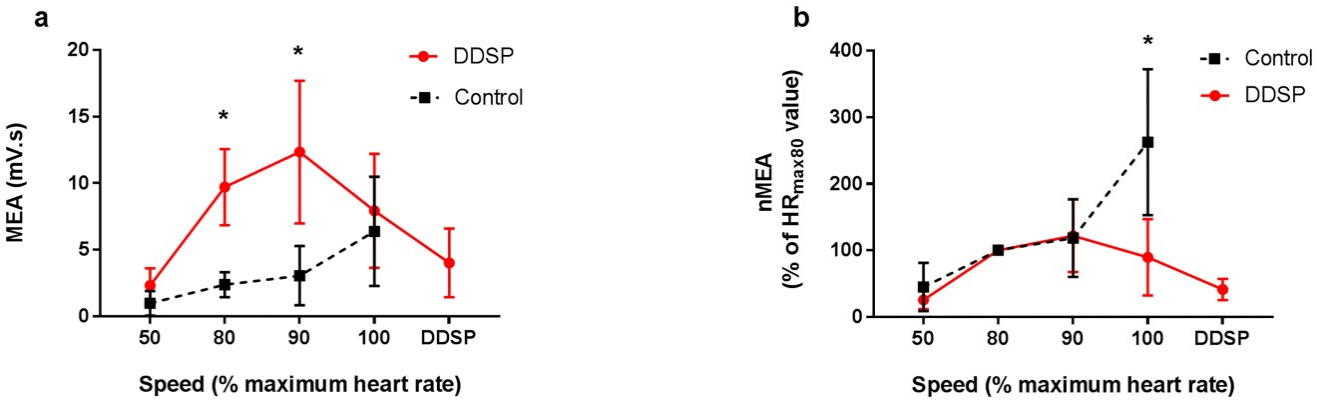
Mean electrical activity (MEA) of the thyro-hyoid muscle decreases at the highest exercise intensity in horses affected by palatal instability and intermittent dorsal displacement of the soft palate (DDSP). 3a) MEA showed significant difference between control and DDSP horses at lower exercise intensity. 3b) MEA normalized to the value recorded at HR_max80_ (nMEA), was significantly lower in horses diagnosed with DDSP compared to control at HR_max100_. Data are expressed as mean with SD. *, p<0.05.

In normal horses, MEA of the TH muscle progressively increased with exercise intensity, reaching the maximum value at HR_max100_ (Fig 3a). The MEA also significantly increased with exercise intensity in the DDSP horses but did so at submaximal exercise intensity (HR_max90_). MEA dropped significantly at the highest exercise intensity in DDSP horses, reaching a minimum just before the horses developed displacement of the soft palate (Figs 2b and 3a). The value calculated just before displacement in two horses are represented in the figures and following the HR_max100_ interval only for graphic purpose. Significant difference was found between control and DDSP horses comparing the normalized MEA (nMEA, normalized to HR_max80_), with a significantly lower nMEA at HR_max100_ in the affected group (p = 0.0215, two-tailed *t*-test, Fig 3b). nMEA dropped to less than 50% of the value recorded at HR_max80_ in two horses just before displacement of the soft palate. No significant asymmetry was detected in MEA and nMEA in either group of horses.

#### Median Power Frequency (MF)

**Fig 4:**
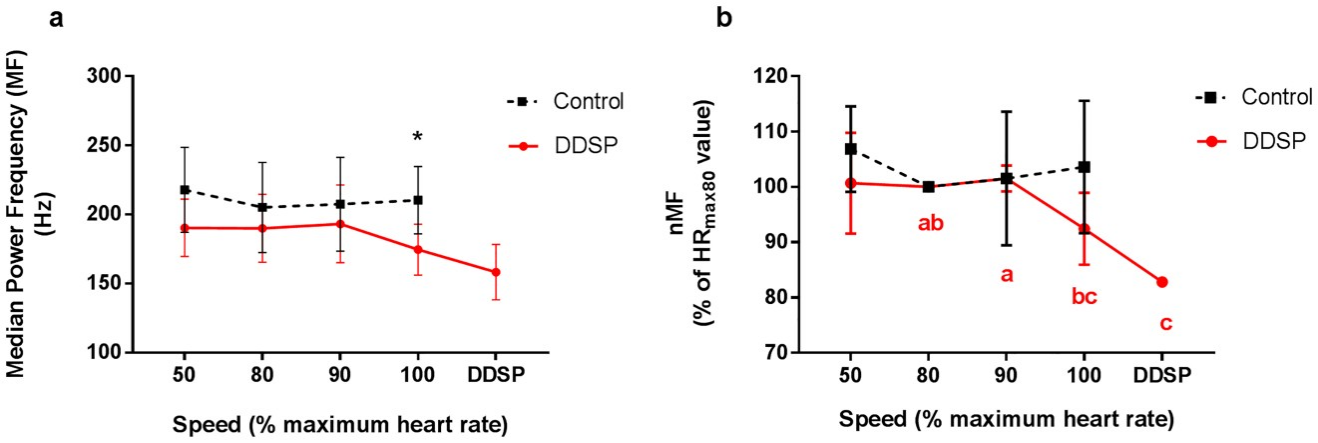
Median Power Frequency (MF) of the thyro-hyoid muscle electrical activity decreases at highest exercise intensity in horses affected by palatal instability and intermittent dorsal displacement of the soft palate (DDSP). 4a) The DDSP group horses show a significantly lower MF at HR_max100_ compared to normal horses. 4b) A significant drop in MF normalized to the frequency recorded at HR_max80_ (nMF) was recorded before developing palatal displacement in 2 horses. Data are mean and SD. * p<0.05. Different letters indicate statistically significant difference between nMF recorded at different speed intervals in the DDSP horses.

A significant difference in MF was found between groups at the highest exercise intensity interval (HR_max100_), with higher value in the control horses (210.2 ± 24.36 Hz) compared to the DDSP horses (174.51 ± 18.46 Hz) (p = 0.0410, two-tail *t*-test, Fig 4a). The two horses that developed displacement of the soft palate, showed a significant drop in MF before displacing the palate (from 193.15 ± 28.04 Hz at HR_max90_ interval to 158.24 ± 19.93 Hz) (p = 0.0125, Tukey-Kramer, Fig 4b). A significant asymmetry in MF was found in the control group, with the left side showing higher MF at the HR_max100_ interval (232 Hz vs 152 Hz of the right TH, p = 0.0309, one-tail paired *t*-test). This asymmetry should be considered cautiously, because bilateral data was available in the control group only from two horses; and no significant asymmetry was detected after normalization (nMF), which allows for correction of difference due to electrode position within the two muscles.

#### Cluster analysis

**Fig 5:**
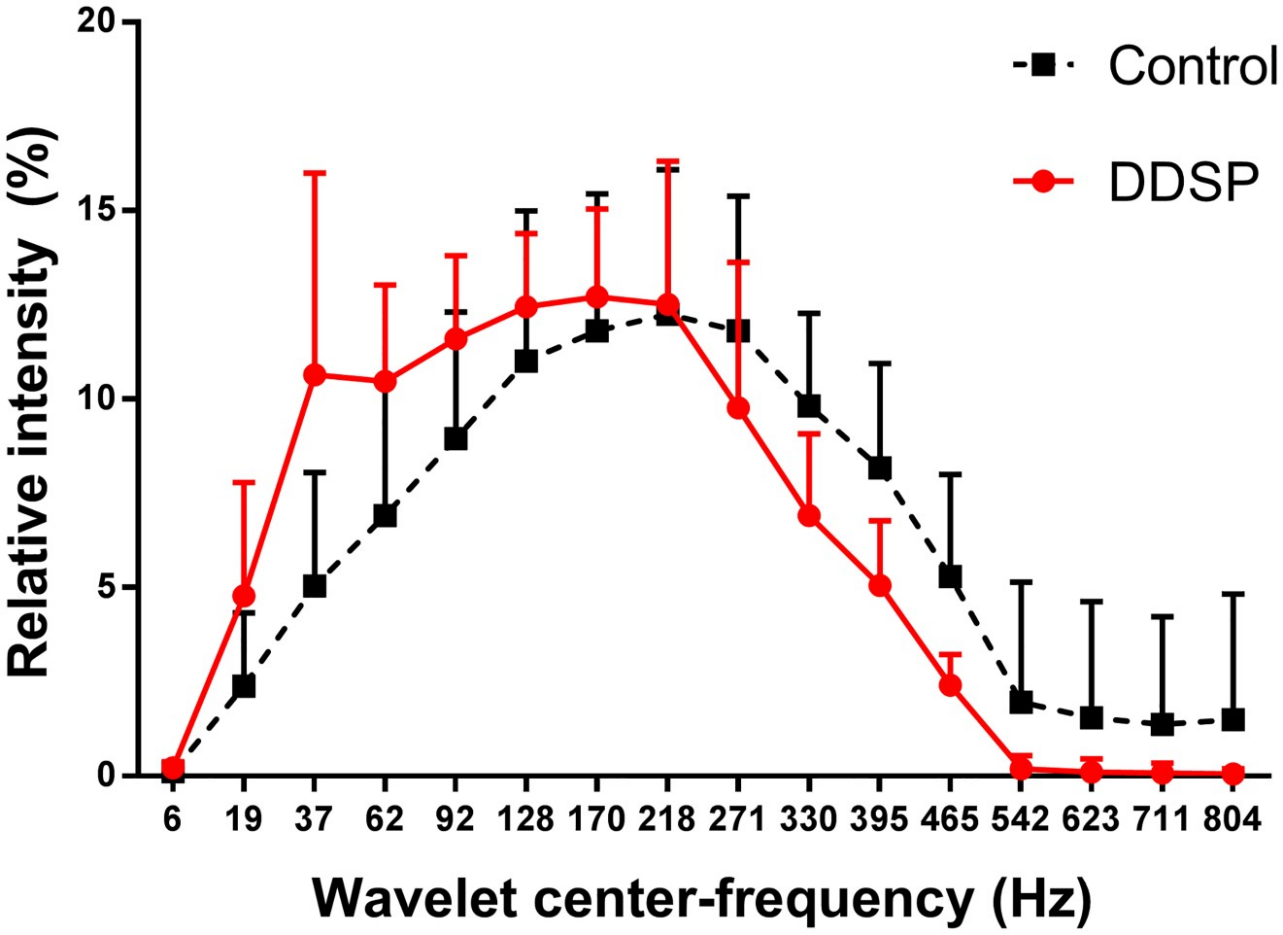
Wavelet decomposition of the thyro-hyoid muscles electromyographic activity in exercising horses. Relative contribution (% of the total intensity) of the center frequency of the 16 wavelets to the thyro-hyoid muscles electromyographic activity shows a relative shift towards lower frequencies in horses affected by palatal instability and intermittent displacement of the soft palate (DDSP) compared to normal horses (control). Data expressed as mean ±SD.

**Fig 6:**
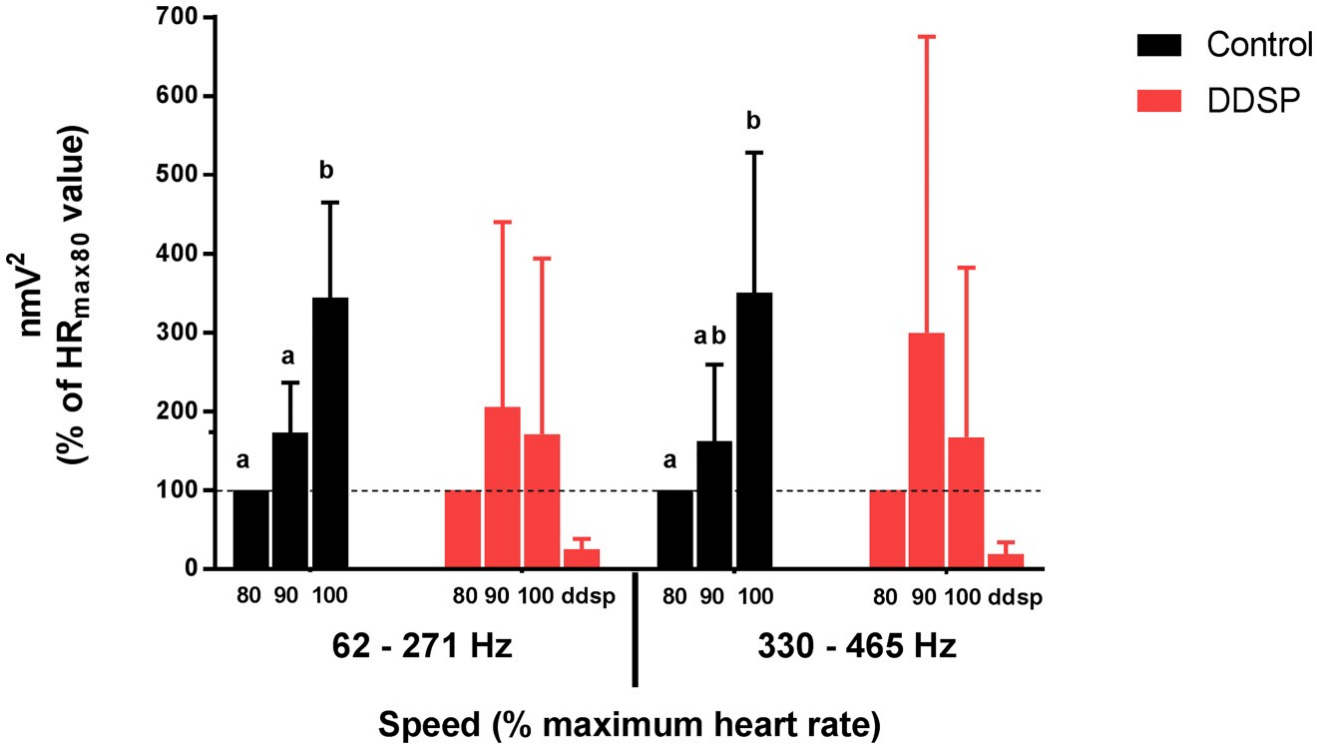
Exercise induced variation in intensity of the two frequency domains identified after wavelet decomposition of the thyro-hyoid muscles electrical activity during exercise. The control group (black bars) showed a significant increase in the intensity of the two frequency domains during exercise, reaching the maximum value at HR_max100_. The DDSP group (red bars) showed a non-significant initial increase followed by a non-significant decrease in the intensity of both domains during exercise. Intensity of each frequency domain is normalized to the value recorded at exercise intensity corresponding to HR_max80_ (nmV^2^). Data are expressed as mean ±SD. Different letters indicate statistically significant difference between intensity values recorded at different speed intervals.

On wavelet decomposition, the EMG traces of both DDSP and control groups showed the majority of the signal contained in the frequency range 62–465 Hz (Fig 5). Within this frequency range, hierarchical agglomerative cluster analysis identified two frequency clusters in the EMG signal, a low-frequency (62–271 Hz) and a high-frequency (330–465 Hz) cluster. The low-frequency cluster contained 68.03 ± 8% of the total intensity of the EMG signal, while the high-frequency cluster contained 18.9 ± 7.2%. On mixed effects model, a significant interaction between group (DDSP vs Control) and exercise intensity (p = 0.0393) was detected on the intensity of the high-frequency (330–465 Hz) domain, and tendency for an effect of the same interaction on the low-frequency domain intensity (p = 0.0533). The control group showed a significant increase in the total intensity for the two frequency clusters during exercise, while the DDSP group data presented high variance, allowing only to infer a trend characterized by a non-significant initial increase followed by a non-significant decrease in the intensity for both clusters (Fig 6).

**Fig 7:**
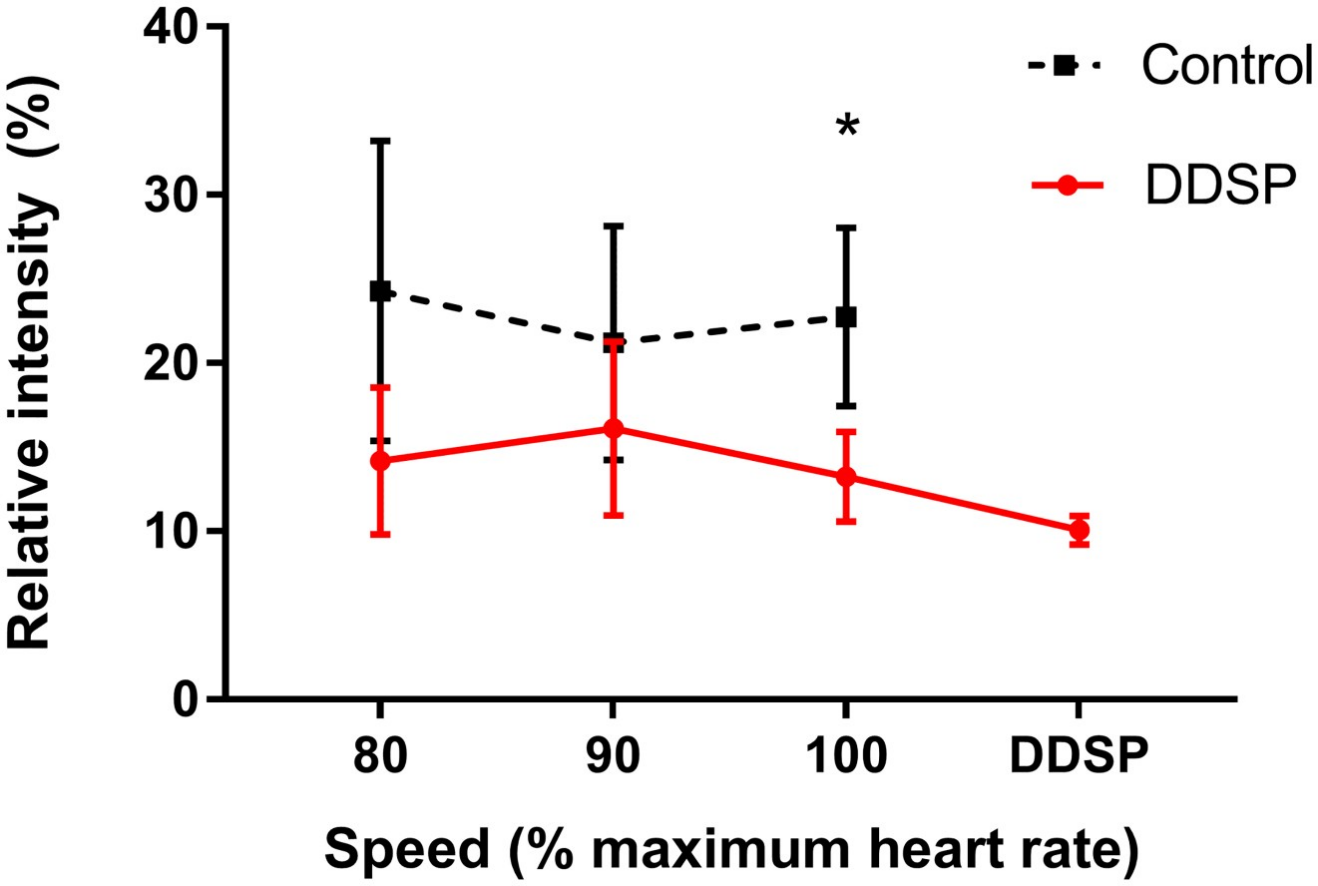
Relative contribution (% of the total intensity) of the high frequency (330–465 Hz) domain to the thyro-hyoid muscles electromyographic activity during exercise. Horses affected by palatal instability and dorsal displacement of the soft palate (DDSP) show a significant lower contribution of the high frequency wavelet domain at the highest exercise intensity interval (HR_max100_). Data expressed as mean ±SD. *, p<0.05.

**Fig 8:**
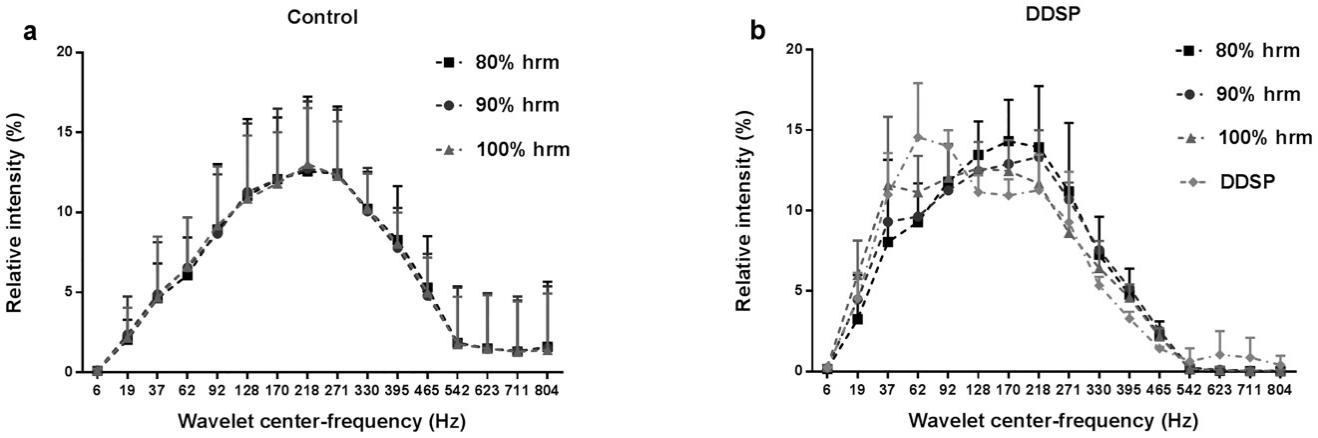
Relative contribution (% of the total intensity) of the 16 wavelets to the thyro-hyoid muscles electromyographic activity during each exercise intensity interval. 8a) Normal horses (control) showed a uniform contribution of each wavelet throughout the exercise. 8b) Horses affected by palatal instability and intermittent dorsal displacement of the soft palate (DDSP), showed a progressive decrease in the contribution of the high frequency wavelets with shift toward a higher low frequency wavelets contribution at highest exercise intensity (100% HRM), and minimum of high frequencies contribution in two horses in the interval preceding the displacement of the soft palate (DDSP). Data expressed as mean ±SD.

The relative contribution to the total intensity of the EMG signal from the two frequency clusters was different between groups, with the control horses having a higher contribution from the high-frequency cluster at the highest exercise level (p = 0.0126, two-tail *t*-test, Fig 7). Fig 8 shows the wavelets relative intensity by exercise intensity, where the control group showed each wavelet contributing uniformly throughout the exercise. The DDSP group, instead, during exercise, showed a progressive decrease in the contribution of the high frequency wavelets with a shift toward low frequency wavelets at highest exercise intensity (100% HRM), and minimum of high frequencies contribution in two horses in the interval preceding the displacement of the soft palate.

Both groups presented asymmetric contribution of the high-frequency cluster to the total intensity of the EMG signal. The control horses showed a higher contribution of the 330–465 Hz cluster on the left TH compared to the right at the intervals HR_max80_ (24.3% vs 15.5%, p = 0.0172, two-tailed paired *t*-test) and HR_max90_ (24.6% vs 11.7%, p = 0.0259, one-tail paired *t*-test). The opposite was detected in the DDSP group with a higher contribution of the high-frequency cluster in the right TH at HR_max90_ (16.8% vs 11.5%, p = 0.0451, two-tailed paired *t*-test). Considering the limited number of horses with bilateral complete dataset, this asymmetry should be interpreted carefully.

## Discussion

This study demonstrates a significant reduction in myoelectrical activity of the TH muscles during exercise in horses affected by naturally-occurring palatal instability and DDSP compared to normal horses. The MEA of the TH muscle progressively increases with exercise intensity in normal horses, indicating increasing muscle fiber recruitment with increasing workload, and reaches maximum intensity at HR_max100_. In contrast, horses affected by palatal dysfunction, with or without DDSP, showed an initial increase, followed by a drop in the myoelectrical activity of the TH muscles at the highest exercise intensity, reaching a minimum in two horses just prior to the development of dorsal displacement of the soft palate. To our knowledge, this is the first study investigating the etiology of DDSP in naturally affected horses, while previously the condition was studied through experimental models [8–10].

The reason for this decrease in TH muscle recruitment in the DDSP horses is unclear but one potential mechanism could be early fatigue, with TH muscle exhaustion at high intensity workload. Associated with the drop in MEA, the two horses that developed displacement of the soft palate also showed a significant drop in median frequency of the EMG power spectrum before displacing the palate. In skeletal and respiratory muscles, EMG signal amplitude and MF increase progressively with increasing force, due to progressive recruitment of additional motor units, increase in their firing frequency and synchronization between MU firing patterns [16,33–38], while a decrease in both amplitude and power frequency is characteristic of fatigue [19].

The traditional power spectrum analysis has limitations in the evaluation of EMG signal during dynamic contraction, because of fluctuations of the spectral parameters due to nonstationarity of the signal. To address this challenge, the wavelet transform was introduced, and found to have better accuracy than the traditional approaches [39]. Wavelet analysis also allows identification of high- and low-frequency bands, proposed to correspond to fast and slow twitch fiber activity, within the myoelectric spectra of different muscles and species [40–42]. Cluster analysis of the wavelet decomposed EMG signal from the TH muscles in this study identified two frequency clusters: a low-frequency cluster including 62–270 Hz frequencies, and a high-frequency cluster with frequencies ranging from 330 to 465 Hz. The evaluation of the trends of the intensity of the two bands during exercise confirmed a significant difference between groups, and allowed to attribute such difference to the high-frequency component of the signal, with the DDSP group experiencing a decrease in the high-frequency band contribution to the EMG signal at the highest exercise intensity. This finding confirms the shift to lower frequency of the MF and allows attributing it to a relative reduction in the high-frequency component supporting the deduction that the changes in the EMG signal in horses affected by palatal instability and DDSP represent muscle fatigue.

In the horse population evaluated in this study, the power spectrum analysis seems to indicate that the TH muscle is mainly composed by type II fibers, leading to median frequency > 150Hz throughout the exercise [17]. The drop in the high-frequency wavelets contribution at the highest exercise intensity recorded in the DDSP group, support the hypothesis that in these horses the fast twitch fibers develop fatigue faster than in control horses.

The predominantly inspiratory activity of the TH muscle has been previously reported in dogs [43]. During exercise, the critical function of the TH muscles seems to be drawing the larynx rostrally and dorsally [9], to counteract the tracheal pull and preventing laryngeal descent. Tracheal pull is the force, generated by the activity of the diaphragm as well as by passive forces during inspiration, which pulls the cricoid cartilage and consequently the larynx, caudally [44]. Fluoroscopic investigation of laryngopharyngeal movement of horses at gallop [45], support that the trachea is extended and “pulled” into a straight position during protraction phase of the stride, phased with inspiration [35]. The same investigation revealed increased rostro-caudal and dorso-ventral excursions of the larynx associated with episodes of DDSP. These excursions support the apparent repetitive caudal retraction of the larynx preceding DDSP that has been clinically noted during endoscopic exam [46]. Combining the aforementioned observations and the results of this study, we hypothesized that in naturally affected DDSP horses, at high exercise intensity, the TH muscle fatigues, leading to failure in moving and stabilizing the larynx rostrally towards the hyoid bone when pulled by the trachea (and presumably strap muscles) during inspiration. Failing this support, the larynx will experience passive rostro-caudal movements (i.e. laryngeal descent), so losing its stable relationship with the caudal border of the soft palate.

The increased fatigability of the TH muscle can either be an intrinsic problem of the muscle (physiological or neuromuscular), sub-optimal fiber type distribution, small size muscles fibers, or sub-optimal training methods for the upper airway musculature. Alternatively, it may develop as a consequence of other upper airway dysfunctions (palatal billowing, other upper airways obstruction, laryngo-hyoid anatomic relationship) that cause an overload of the TH muscle during exercise leading to its fatigue.

Fatigue is defined “a loss in the capacity for developing force and/or velocity of a muscle, resulting from muscle activity under load and which is reversible by rest” [47]. Two main mechanisms have been identified in the development of muscle fatigue: ‘‘central fatigue” as a decrease in the recruited motor units (MU) number and discharge rates, and ‘‘peripheral fatigue” indicating a decrease in the transmission of muscle action potentials and contractile strength of the muscle fibers [48]. The reasons for fatigue to occur in the TH muscle are unknown but could be a different fiber-type proportion of the TH muscles in horses affected by DDSP: that is a reduction in fatigue resistant type I muscle fibers. Muscle with a high prevalence of fiber type I are characterized by slow contractility but higher fatigue resistance, thanks to a higher content and activity of oxidative enzymes in these fibers [49]. A higher percentage of type II fibers makes the muscle a fast contractor but more vulnerable to fatigue due to accumulation of metabolites, like reactive oxygen species (ROS) [50,51]. ROS accumulation is responsible for depression of force production and increased fatigue in skeletal muscle; marked differences exist between muscle fiber type regarding production and metabolism of ROS, with fiber type I showing higher activities of anti-oxidant enzymes and consequently higher scavenging capacity than type II [51].

All horses included in the DDSP group, were confirmed displacers during 3 overground tests at the time of recruitment to the study. After implantation of the intramuscular electrodes and training on the treadmill, two of these horses experienced palatal instability but did not show any episodes of palatal displacement during repeated tests on the treadmill. It is possible that the overground protocol subjected the TH muscles to a higher workload (higher peak speed, faster peak speed reach, longer distance), inducing DDSP faster, while during the test on treadmill they never reached the threshold point for DDSP to happen. Unlike the other horses included in the study, due to logistic difficulties, these two horses were not tested during an incremental exercise test on treadmill prior to surgery. The lack of pre-operative data during an incremental exercise on treadmill and/or post-operative overground test from these two horses is a limitation, because it could have clarified the effect of exercise protocol on the occurrence of palatal displacement in these horses.

It has been reported that DDSP is diagnosed more frequently during treadmill tests than overground endoscopy, with a larger proportion of horses during the treadmill tests that progressed from palatal instability to DDSP [52]. For this reason, to explain the lack of DDSP episodes in two of our horses after instrumentation, beside the different exercise protocol, we considered also other possible mechanims, related to the surgical procedure and to time and training. The lack of DDSP episodes in these two horses could result from a change in the muscle structure due to the surgical procedure (fibrosis) leading to a more stable laryngo-hyoid relationship or an initial different cause of DDSP that improved or resolved after surgery (ie. lower airway inflammation).

However, the analysis of the EMG traces in both horses with palatal instability but no longer displacing was also indicative of TH fatigue despite the different outcome. This is consistent with clinical reports that considers palatal instability with or without DDSP, manifestations of the same condition, palatal dysfunction [31,32]. There is evidence that many horses show DDSP at the beginning of their carrier, during the initial training period, possibly associated to an ‘immature nasopharynx”, that spontaneously improves or resolves with time, in correlation with continued growth or maybe in association with the training itself [53–55]. A possible explanation of this reported spontaneous resolution, and the lack of DDSP episodes in our two horses could rely in improvement of the TH muscle fatigue resistance, with raising of the fatigue threshold for DDSP to occur, obtained with the regular training.

The positive effect of training exercise on muscle fatigue resistance and electromyographic characteristics has been well described, with a slower decrease in median frequency found in trained subjects [18]. Endurance training has been reported to increase inspiratory muscle strength and fatigue resistance already after 3 weeks [56]. While lower intensity exercise increases the mitochondrial volume in type I fibers, much higher intensity exercise recruits mitochondria in type II fibers [51,57]. Unfortunately, we are lacking objective data like pre-training electromyographic recordings or muscle histologic assessment, which would have helped in characterizing the muscle condition at the time of enrollment in the study and at the end of the study.

The main limitation of this study is the small sample size, only four horses with naturally occurring disease and only two that actually displaced after instrumentation. Although the lack of palatal displacement in these two horses represents one potential pitfall of this study, it gave us the possibility to evaluate horses with the two manifestations of naturally occurring palatal dysfunction. It is plausible that the two horses with palatal instability but no longer showing displacement, improved their TH fatigue resistance through training, a valid hypothesis also for the spontaneous resolution detected in some horses with DDSP reported by Pollock et al. [55].

The placement of intramuscular electrodes connected to a telemetric system is a novel methodological approach, which allowed multiple EMG recordings during high-speed exercise trials in some of the horses. Evaluation of TH electromyographic activity during exercise in a larger number of horses naturally affected by DDSP would be ideal. However, due to the invasive nature of the implantations and the fact that is very difficult to perform non-invasive measures of the TH muscle during exercise, investigation on a larger number of horses is unlikely with the current approach.

Further studies are required to confirm that peripheral fatigue is the sole responsible of the TH dysfunction leading to DDSP, and to evaluate the fiber type composition and metabolic characteristics of the TH muscle in normal and DDSP affected horses. The consequent step would be to evaluate the effect of different types of training protocol on TH muscle characteristics to identify a new therapeutic approach for DDSP prevention and cure. The positive effect of endurance training exercise on muscle fatigue resistance has been well described, with an increase in number of capillaries, activity of oxidative enzymes, mitochondrial biogenesis and changes in muscle fiber type composition [18,51,57], and increased inspiratory muscle strength and fatigue resistance [56,58,59].

## Conclusions

In this study, we identified fatigue as the main factor leading to palatal instability and exercise induced dorsal displacement of the soft palate in a group of racehorses, based on electromyographic activity of the thyro-hoid muscles. The cause of this increased fatigability in the TH muscles in some horses still needs to be identified, and histologic assessment of TH muscles in DDSP-affected horses would be the next step. Confirmation of different TH muscle characteristics (fiber type composition, metabolic features) in DDSP-affected horses would open the possibility to use muscle reconditioning through specific training as a non-surgical option for the management of DDSP in young racehorses.
